# A demand-centered scheduling framework for shared supercomputing resources: modeling, metrics, and case insights

**DOI:** 10.1038/s41598-025-02353-9

**Published:** 2025-05-22

**Authors:** Hyungwook Shim

**Affiliations:** Korea Institute of Science and Technology Information, Daejeon, South Korea

**Keywords:** Supercomputing, Joint utilization system, Dynamic scheduling, Demand management, Resource efficiency, Energy science and technology, Mathematics and computing

## Abstract

The exponential growth of artificial intelligence and data-intensive applications has led to a significant surge in demand for supercomputing resources. However, limited infrastructure capacity and rising construction costs have made traditional supply-side expansion strategies increasingly unsustainable. In response, many nations are exploring joint utilization systems to maximize resource efficiency by enabling the flexible allocation of distributed computing assets. This study proposes a novel dynamic scheduling framework designed to enhance demand-side management in such environments. The methodology involves estimating a demand model using price elasticity analysis and developing a new composite index to quantitatively evaluate resource management efficiency across multiple centers. A comparative case study was conducted using simulated data from seven specialized supercomputing centers, analyzing different scheduling strategies under varying joint resource ratios. To verify the effectiveness of the proposed framework, an additional comparative analysis was performed for three organizations under identical resource conditions. The results reveal that the dynamic scheduling method provided up to 3.5 times more effective average resource delivery compared to the static method. Furthermore, while the static scheduling method resulted in a response failure rate exceeding 30%, the dynamic method reduced this to approximately 8%, clearly demonstrating its superior ability to meet fluctuating demands with the same amount of resources. These results demonstrate that the proposed dynamic scheduling method significantly reduces demand-response failures and surplus idle resources compared to conventional static scheduling. Furthermore, the study introduces a system-wide efficiency index, which enables real-time monitoring of temporal and institutional demand variance. These findings provide both theoretical and practical contributions to the design and governance of shared HPC infrastructures. The proposed approach offers a scalable foundation for policy frameworks and operational strategies in multi-institutional supercomputing environments.

## Introduction

In recent years, the demand for high-performance computing (HPC) resources has grown dramatically due to the widespread adoption of artificial intelligence (AI), big data analytics, and simulation-driven research across scientific and industrial domains. Traditionally, HPC infrastructure strategies have prioritized the construction and expansion of physical supercomputing systems, with the assumption that computational capacity is directly correlated with national scientific and technological competitiveness. However, this supply-side approach is increasingly challenged by soaring construction costs, hardware limitations, and the widening gap between demand and available resources—especially in countries lacking domestic supercomputer manufacturing capabilities. To address these issues, a paradigm shift toward more efficient utilization of existing resources is emerging. One promising approach is the implementation of joint utilization systems, which consolidate distributed supercomputing resources and enable flexible allocation across institutions. This system allows organizations to share computational capacity dynamically, thereby reducing the need for continuous infrastructure expansion and improving national-level responsiveness to fluctuating demand. Several countries have begun exploring governance frameworks for such shared systems, yet systematic research on effective operational strategies remains scarce. Current resource management methods in many HPC environments are predominantly supply-oriented, involving fixed-time allocations or reservation-based usage models. While these methods allow for structured planning, they lack flexibility and often result in inefficient use of resources when user demand deviates from expected patterns. Moreover, existing studies tend to focus on single-institution scheduling or resource allocation from the provider’s perspective, neglecting demand variability and system-wide optimization.

Against this backdrop, the present study proposes a new operational model for joint utilization systems, emphasizing dynamic scheduling to support demand-side resource management. By leveraging demand forecasting models and introducing a novel efficiency index for multi-institutional coordination, this research aims to improve the adaptability and responsiveness of supercomputing resource governance. Through empirical simulations and comparative evaluation of multiple scheduling scenarios, the proposed model seeks to offer both theoretical insight and practical guidance for future HPC infrastructure planning.

## Literature review

As the demand for HPC surges with the exponential growth of data-intensive and latency-sensitive applications, the efficient management of distributed and heterogeneous computational resources has become a critical research challenge. In particular, the emergence of joint utilization systems, dynamic scheduling algorithms, and demand-side management approaches reflect a growing recognition that traditional static resource allocation methods are insufficient in contemporary multi-institutional and distributed computing environments. This literature review aims to examine recent research that informs and supports the development of a demand-centered dynamic scheduling framework, especially in the context of shared supercomputing infrastructures. The selected studies were chosen based on their methodological relevance, their focus on elasticity, dynamic resource allocation, and reinforcement learning applications in HPC, and their contributions to overcoming limitations of current resource scheduling approaches.

Liu (2018) proposes a series of elastic scheduling approaches to address key challenges in HPC resource management, including long response times in batch scheduling systems, low utilization in on-demand clusters, and complexity in scheduling across heterogeneous resources. The proposed techniques—Elastic Job Bundling (EJB), Balancer, and Bundle—enable dynamic resource allocation and non-invasive integration into existing systems. Simulation and real-world experiments demonstrate significant improvements: EJB reduces turnaround time and resource fragmentation; Balancer minimizes the need for dedicated on-demand clusters while improving wait times; and Bundle offers a unified abstraction for managing heterogeneous HPC environments^1^. Goudarzi (2020) proposes a novel three-tier edge computing (EC) framework designed to enhance real-time vehicular monitoring by dynamically allocating computational resources across cloud, edge, and device layers. To address the latency constraints of cloud-based vehicular networks, the authors integrate EC with software-defined networking (SDN) to form an SDN edge (SDNE) system. The resource allocation challenge is formulated as an optimization problem and tackled using a multi-agent reinforcement learning (RL) algorithm enhanced by experience replay. The proposed method enables adaptive routing and elastic processing capacity assignment to suitable edge servers, leveraging real-time communication and network state data. Simulation results and a real-world case study demonstrate the framework’s effectiveness in improving computational efficiency and responsiveness in autonomous vehicle networks^2^. Souza (2021) introduces an adaptive reinforcement learning-based co-scheduling algorithm for HPC environments, aimed at addressing the inefficiencies arising from limited visibility into application-level resource usage. By combining application profiling with real-time cluster monitoring, the proposed scheduler dynamically matches resource utilization to actual application demands at the OS level. Unlike traditional nominal allocations, the algorithm employs decision tree models to estimate resource co-allocation opportunities, continuously learning from scheduling missteps and adapting to system dynamics. Integrated with a hybrid resource management system based on Slurm and Mesos, the method demonstrated significant improvements—up to 51% in cluster utilization and 55% reductions in job queue makespan—across a diverse set of real scientific workflows, particularly under varying workload conditions^3^. Abhishek (2020) presents a dynamic resource allocation model aimed at addressing the persistent challenges of resource usage and performance overhead in HPC clusters. Recognizing the increasing demand for fast computation in today’s data-driven environment, the authors propose a parallel, interconnected system architecture to support supercomputing functionalities. The model focuses on the dynamic assignment of CPU and memory resources to improve execution efficiency. Experimental results demonstrate how varying configurations of computing resources and process interconnectivity affect execution time, offering insights into optimal cluster resource management strategies^4^.

Across these studies, several key themes emerge: the need for dynamic, elastic resource scheduling mechanisms in HPC environments; the growing role of reinforcement learning in optimizing resource allocation; and the value of integrating network-aware or architecture-aware layers such as SDN or application profiling to improve efficiency. The reviewed literature highlights that static or supply-side focused strategies are increasingly inadequate in addressing the real-world complexity of distributed and heterogeneous supercomputing systems. These findings collectively validate the motivation for developing a demand-centered scheduling framework tailored for shared supercomputing resources. While previous studies have tackled related issues—such as resource fragmentation, scheduling latency, and workload prediction—there remains a gap in systematically integrating demand-side modeling with scheduling policies in multi-institutional HPC infrastructures. Therefore, this study builds upon the foundations laid by prior work to propose a simulation-based scheduling framework that incorporates demand elasticity, differentiated user responsiveness, and real-time policy evaluation. By addressing both theoretical and practical dimensions of supercomputing governance, this research aims to offer a scalable and reproducible foundation for next-generation resource management strategies.

## Operational methods of supercomputer resources

The management of supercomputing resources traditionally follows rigid, supply-centered paradigms, often reflecting institutional control and legacy operational structures. These approaches can be broadly categorized into three principal models based on their allocation mechanisms and usage contexts (Table 1). The first model is a batch-based allocation system, in which supercomputing resources are distributed to selected users on a scheduled basis, often three times per year. This model enables predictable resource management and supports large-scale or long-term research projects requiring sustained computational capacity. However, its inflexible nature poses limitations: extensions beyond the allocated period are often infeasible, and unexpected demand surges cannot be accommodated, potentially interrupting ongoing research activities. The second model involves continuous or on-demand access, where users are granted resources whenever baseline computational capacity is available. This model provides more temporal flexibility but suffers from unpredictability in resource availability, making it challenging for users to plan large-scale computational tasks with confidence. The third model addresses urgent national priorities, such as weather forecasting, natural disaster response, or pandemic simulations. In these cases, resources are forcibly reallocated—preempting existing jobs to prioritize critical missions. While essential in emergencies, such ad hoc reallocations underscore the vulnerability of current systems to unexpected demands and raise questions about equity and long-term scheduling reliability^5–7^.

Collectively, these models highlight a critical shortcoming: all are fundamentally supply-oriented. They focus on managing access to limited resources based on predefined institutional priorities rather than responding to user-side demand patterns. As AI, data science, and simulation-intensive research continue to proliferate, this supply-side rigidity increasingly hampers scientific productivity and resource efficiency. To mitigate this, several countries are implementing joint utilization systems, which aim to integrate distributed supercomputing resources across multiple institutions into a cohesive, nationally-coordinated framework. These systems often leverage high-speed national networks and cloud-based architectures to enable flexible resource sharing. Users with demand exceeding their local resource allocations can temporarily access pooled national resources, thereby improving overall utilization and responsiveness. However, such systems require new operational paradigms. Merely pooling resources without adaptive allocation mechanisms risks reproducing existing inefficiencies at a larger scale. Effective implementation demands not only integrated infrastructure but also robust governance models and demand-responsive scheduling algorithms. To this end, there is a pressing need for demand-centric resource management strategies that reflect temporal variation, workload heterogeneity, and user preferences.

**Table 1:** Supercomputing resource management models.

Model	Description	Strengths	Limitations	Use Cases
Batch-based Allocation	Resources are allocated on a fixed schedule (e.g., 3 times/year) for long-term projects.	- Predictable resource usage - Suitable for large-scale research	- Inflexible - Cannot extend or adapt to sudden needs	Long-term scientific projects
Continuous/On-Demand Access	Resources are allocated whenever available, based on current system load.	- Flexible in time - Enables opportunistic computing	- Unpredictable availability - Not suitable for planned large-scale jobs	Short interactive tasks, opportunistic computing
Urgent National Priority Reallocation	Resources are forcibly reassigned for emergencies, interrupting other tasks.	- Supports critical missions - Prioritizes national interests	- Disruptive - Raises equity and reliability concerns	Disaster response, pandemic simulations, forecasting

However, such systems require new operational paradigms. Merely pooling resources without adaptive allocation mechanisms risks reproducing existing inefficiencies at a larger scale. Effective implementation demands not only integrated infrastructure but also robust governance models and demand-responsive scheduling algorithms. One such operational innovation is the introduction of dynamic resource scheduling—a strategy that enables real-time reallocation of idle or underutilized computing capacity based on current demand and system status. Unlike static scheduling, which grants users fixed allocations regardless of actual usage, dynamic scheduling identifies and redistributes surplus capacity across institutions to prevent resource waste and mitigate demand-response failures. For instance, if an institution holds but does not fully utilize its reserved capacity, other organizations with unmet demand may temporarily access the idle portion without compromising fairness. This approach enhances the overall responsiveness and equity of the system while maintaining stability. As such, dynamic scheduling is a key enabler of scalable, demand-aware resource management in federated HPC environments and will be explored in detail in the subsequent chapters.

## Demand management method for the joint utilization system

As joint utilization systems become increasingly important for balancing supercomputing supply and demand, a tailored demand management strategy is essential to ensure efficient and equitable access to computational resources. Traditional demand forecasting methods, such as trend-based extrapolation and survey-based intention analysis, offer initial insights but fall short of capturing the complexity inherent in multi-institutional supercomputing environments.

### Demand modeling framework

To estimate demand behavior in supercomputing systems, we adopt three representative functional forms commonly used in demand analysis:

1 $$\:\text{V}=\text{a}{\text{P}}^{\text{b}}\:\text{(Non-linear power function)}$$

2 $$\:\text{V}=\text{a}+\text{b}\text{P}\:\text{(Linear model)}$$

3 $$\:\text{V}=\text{a}+\text{b}\text{l}\text{n}\text{P}+\text{c}\text{P}\:\text{(Log-linear hybrid model)}$$

Here, V denotes the volume of demand, and P is the price (e.g., cost per CPU day). Among these, Eq. (4) is selected as the baseline model due to its ability to capture price elasticity behaviors that exhibit diminishing demand sensitivity—an appropriate assumption given that most supercomputing users are publicly funded institutions with relatively inelastic price response. Price elasticity is further derived by differentiating the demand function and simplifying as:

4 $$\:\epsilon\:=\partial\:V/\partial\:P\cdot\:P/V=a\cdot\:b\cdot\:{P}^{b-1}\cdot\:P/a\cdot\:{P}^{b}=b$$

This derivation confirms that elasticity can be interpreted directly from the exponent in the demand function^8,9^.

### Lessons from energy sector demand management

The concept of demand management has long been applied in the electricity sector, where real-time balancing of supply and demand is critical for grid stability and operational efficiency. Key techniques include:

- Peak Clipping: Limiting maximum demand to reduce stress on infrastructure.
- Peak Shifting: Moving demand from peak to off-peak hours.
- Valley Filling: Encouraging increased demand during periods of low usage.

While these strategies offer valuable precedents, their direct application to supercomputing systems is non-trivial. Unlike geographically dispersed power grids, HPC resources in a joint utilization system are cloud-connected and location-independent. Moreover, computational resource allocation can be dynamically adjusted without physical delays, and the marginal cost of utilization is typically lower than in energy systems.

### Proposed demand management model for HPC systems

Given the distinct characteristics of supercomputing infrastructure, we propose a model grounded in structural change indicators such as:

- Total resource capacity.
- User institution profiles.
- Resource utilization history.
- Pricing and policy variables.

Based on these structural variables, we derive four distinct demand management options suitable for a federated HPC environment (see Table 2).

**Table 2:** Demand management options for joint utilization systems.

Option	Resource Ownership	Joint Resource Use	Allocation Priority	Allocation Style
1	Fully Individual	✘	Not Applicable	Not Applicable
2	Partial Joint (Isolated)	✔	None	Not accessible routinely
3	Shared (Prioritized)	✔	First-come, first-served	Proportional to request
4	Shared (Equally Distributed)	✔	First-come, first-served	Equal shares to all

- **Option 1**: Each center operates independently, with no shared resources. While reserve management is easier, it results in high failure rates when demand exceeds local capacity.
- **Option 2**: Some resources are isolated as shared reserves, used only when other centers face shortfalls. This increases overall resilience but reduces day-to-day flexibility.
- **Option 3**: Joint resources are accessible based on request order, allocating varying amounts depending on individual center needs.
- **Option 4**: Requests are granted simultaneously in equal shares, emphasizing fairness over specificity.

### Evaluation criteria and strategy selection

To select the optimal demand management strategy, quantitative evaluation metrics must be applied. In particular, the suitability of each strategy depends on:

- The ability to absorb peak demand.
- The responsiveness to urgent use cases.
- The amount of unused but reserved capacity.
- System-wide load balancing and fairness.

The subsequent sections introduce a set of performance indices—specifically a time-distributed efficiency metric and a dynamic scheduling model—that collectively support a data-driven selection of operational policies in joint HPC resource environments.

## **Development of evaluation index for demand management methods**

As discussed in the previous chapter, in order to introduce demand management, an efficiency measurement indicator is needed that can reflect the characteristics of the demand curve of each individual institution. So far, the only evaluation of operational efficiency for supercomputer resources is using the load factor^10^. It is the same as Eq. (5), where $\:{D}_{Avr}$ represents the daily average demand of a specific institution, and $\:{D}_{Max}$ represents the daily maximum demand.

5 $$\:LF={D}_{Avr}/{D}_{Max}$$

**Fig. 1:**
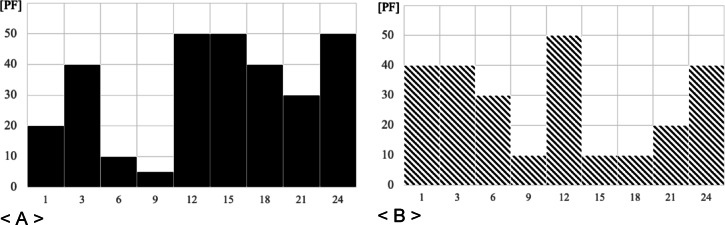
Daily demand curves **A**, **B**.

This index indicates how effectively resources are being used and the level of resource efficiency. Thus, the suppression, movement, and inflow of demand can be determined in a way that increases efficiency. As illustrated in Fig. 1, in the case of institution A, $\:{D}_{Avr}$ is 33 PF and $\:{D}_{Max}$ is 50 PF, which has an $\:LF$ value of 0.66, while institution B has $\:{D}_{Avr}$ of 28 PF and $\:{D}_{Max}$ of 50 PF, and has an $\:LF$ value of 0.56. In other words, institution A has less variation in resource use than does institution B, and the resource utilization rate is relatively high. Additionally, it can be said that organization A is managing its resources about 25% more efficiently than organization B.

However, because this index only evaluates the operational efficiency of a single institution and resource, it is not suitable for a joint utilization system. Therefore, operational efficiency must be evaluated by comprehensively considering multiple organizations. Improvements should be made to increase the efficiency of the overall system through the most ideal resource size (joint utilization ratio) and demand distribution for each field. We developed a new evaluation index in line with this direction of improvement, and the index is as shown in Eq. (6). $\:{\sum\:D}_{Max}$ refers to the sum of the maximum values ​​for each specialized center based on the daily demand curve, and $\:{\sum\:D}_{Max\_t}$ refers to the maximum value for the sum of demand for each specialized center in a specific time period.

6 $$\:{I}_{td}=\sum\:{D}_{Max}/\sum\:{D}_{Max\_t}$$

**Fig. 2:**
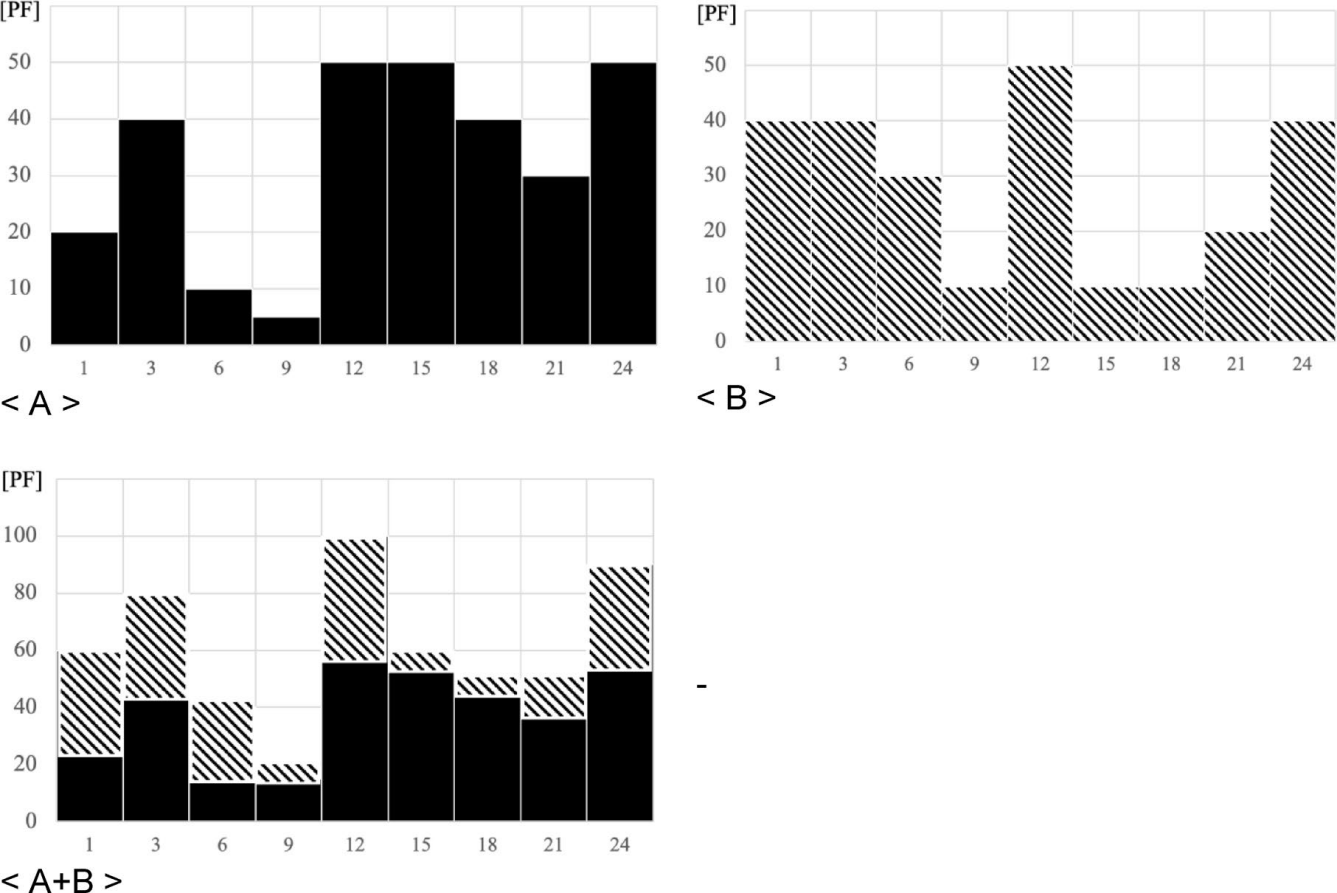
Demand curve example.

For example, as shown in Fig. 2, the $\:\sum\:{D}_{Max}$ of two institutions A and B is calculated by adding the maximum value of 50 PF for institution A and 50 PF for institution B, resulting in 100 PF. $\:{\sum\:D}_{Max\_t}$ is calculated as 100 PF, which is the addition of 50 PF for institution A and 50 PF for institution B in the 12-hour section. Through this, $\:{I}_{td}$ is calculated as 1.0. A value greater than 1, indicates a large fluctuation (time, size) in demand by institution, and a value of 1, indicates that the maximum demand occurs in multiple institutions in the same time zone. Cases less than 1 do not occur. $\:{I}_{td}$, in terms of demand management suggests, that by monitoring this value, the timing and size of demand can be controlled, and through this, multiple resources can be used more comprehensively and efficiently.

## Case study

**Fig. 3:**
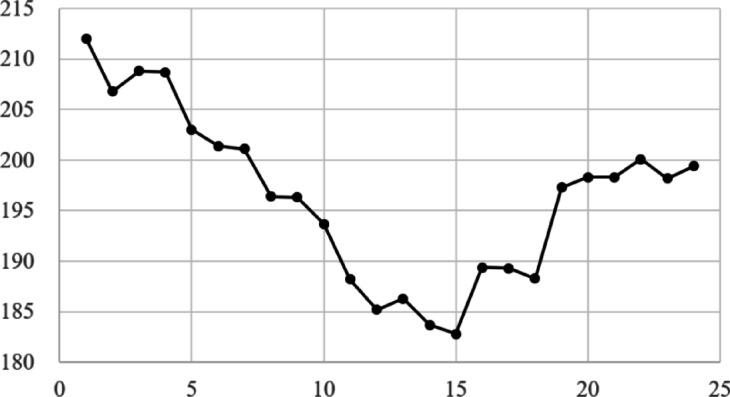
Daily demand curve (total).

**Fig. 4:**
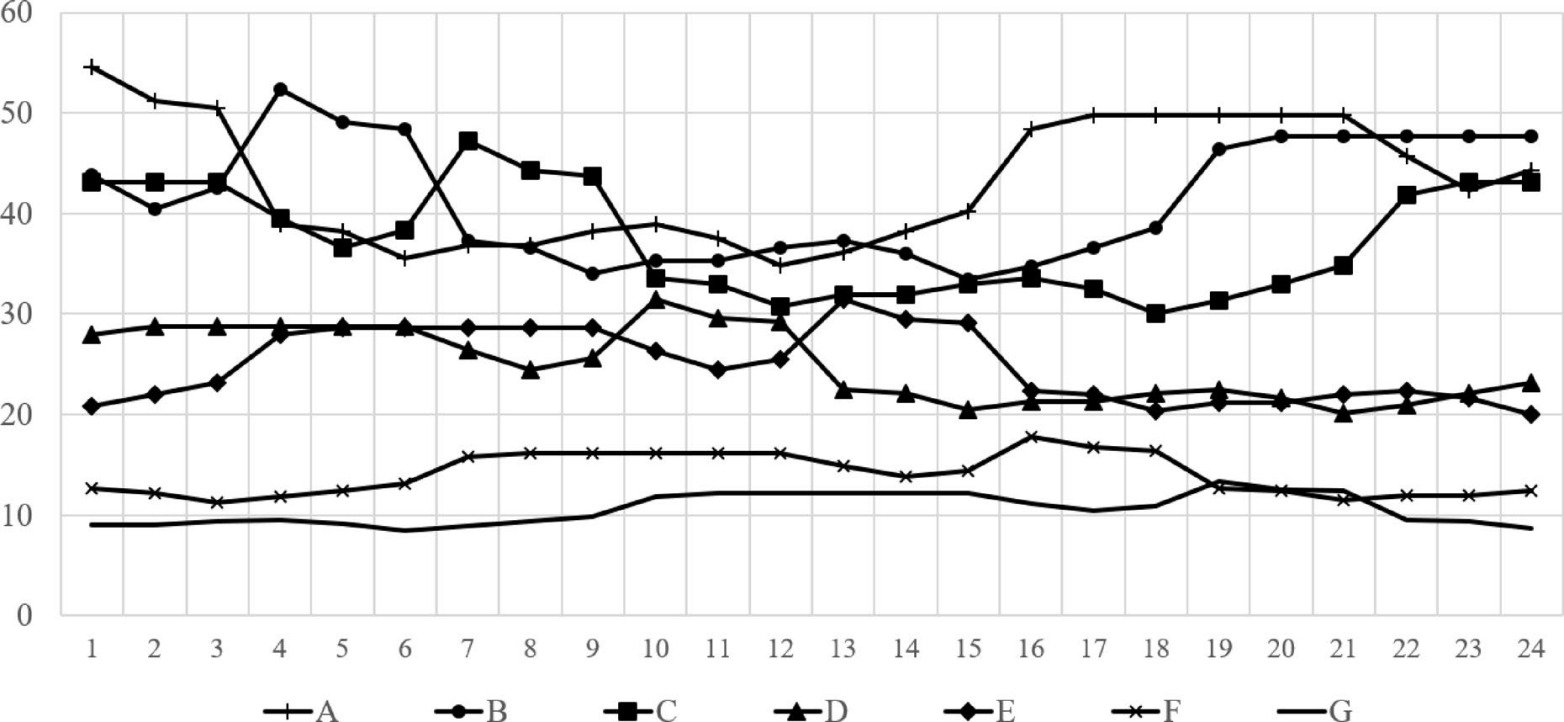
Daily demand curve (specialized center).

A case study was conducted to compare and analyze the effectiveness of demand management methods. It requires an analysis of two situations. First, when operating joint resources, the ability to respond to demand exceeding the limited amount of resources must be examined. To this end, the operational effectiveness when specialized centers individually respond to demand exceeding the resources without joint resources must be compared with that when they respond with joint resources. Operational effectiveness refers to the value of $\:{I}_{td}$, the presence and size of demand response failures. Second, to examine the response ability according to the size of joint resources, the operational effect according to changes in the ratio of joint resources must be examined. As the ratio of joint resources increases, the resources shared with specialized centers in other fields increase. Thus, being able to respond more effectively when the demand for the use of a specific specialized center exceeds the limited resources is advantageous. However, as the marginal resources provided by specialized centers to the relevant field decrease, the demand for regular use inevitably decreases. The operating conditions of the seven specialized centers must be assumed to conduct the case study. Limited to Korea, the daily demand trends of national centers with the most similar resource operation purposes and characteristics were randomly applied to each specialized center. In addition, total resources were assumed to be the sum of the resources each specialized center would like to possess. The daily demand curves of the seven specialized centers are shown in Figs. 3 and 4.

The case study conditions are summarized in Table 3. The joint resource ratio refers to the operational methods of seven specialized centers acquired from the Ministry of Science and ICT and was set considering the maximum value that can be realistically set (60%) based on the 20% value in 2023. To assess the ability to respond to demand that exceeds available resources, the size of the demand injected at a specific time was set within the range of 80–250% of the resources held by the specialized center.

**Table 3:** Conditions of case study.

	Joint resource ratio (%)	Injection demand (PF)	Time (hour)	Note
1	20	40	4	B
2	40	70	10	D
3	60	100	15	F

The resource characteristics of each specialized center, reflecting the case study conditions, are presented in Tables 4, 5 and 6.

**Table 4:** Resource characteristics of specialized center (Joint resource ratio: 20%).

	A	B	C	D	E	F	G
Total (PF)	85.3	81.7	73.7	49.2	49.1	27.7	20.9
Joint resource	17.1	16.3	14.7	9.8	9.8	5.5	4.2
Reserved resource	68.2	65.4	59	39.4	39.3	22.2	16.7

**Table 5:** Resource characteristics of specialized center (Joint resource ratio: 40%).

	A	B	C	D	E	F	G
Total (PF)	85.3	81.7	73.7	49.2	49.1	27.7	20.9
Joint resource	34.1	32.7	29.5	19.7	19.6	11.1	8.4
Reserved resource	51.2	49	44.2	29.5	29.5	16.6	12.5

**Table 6:** Resource characteristics of specialized center (Joint resource ratio: 60%).

	A	B	C	D	E	F	G
Total (PF)	85.3	81.7	73.7	49.2	49.1	27.7	20.9
Joint resource	51.2	49	44.2	29.5	29.5	16.6	12.5
Reserved resource	34.1	32.7	29.5	19.7	19.6	11.1	8.4

**Fig. 5:**
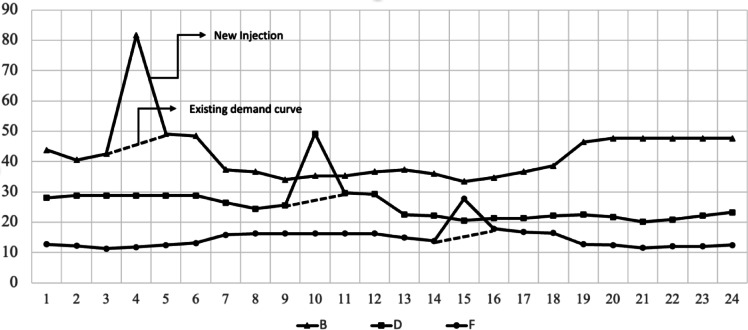
Demand curve (with conditions).

The results of reflecting the case study conditions in the existing daily demand curve are shown in Fig. 5. At a certain point in time, the demand for specialized centers B, D, and F that have been injected with usage demand reaches a peak value that exceeds the limit of resources.

**Fig. 6:**
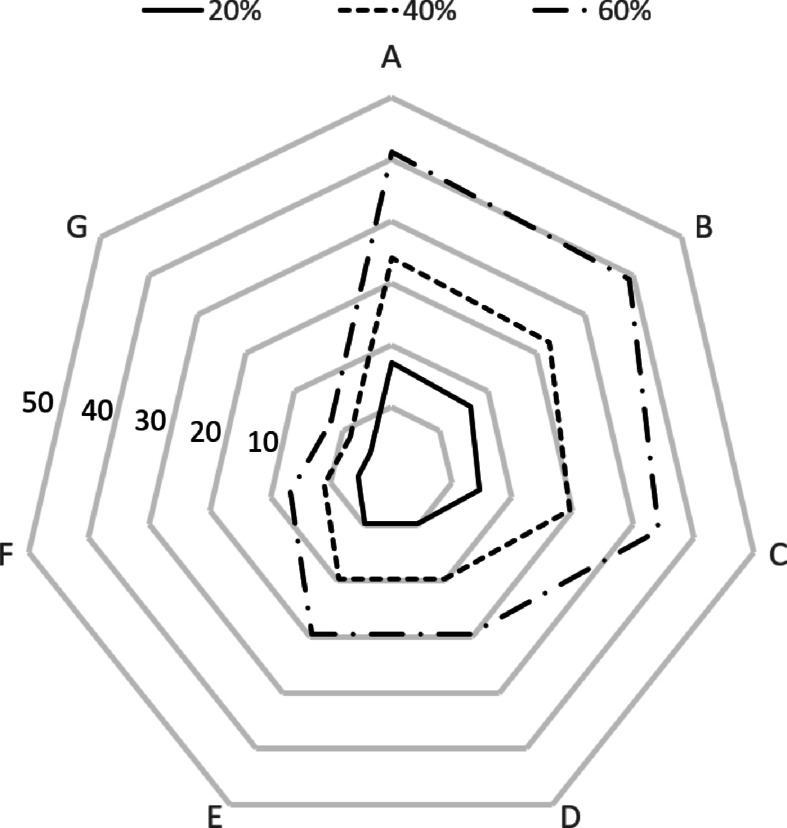
Analysis results (joint resources).

**Fig. 7:**
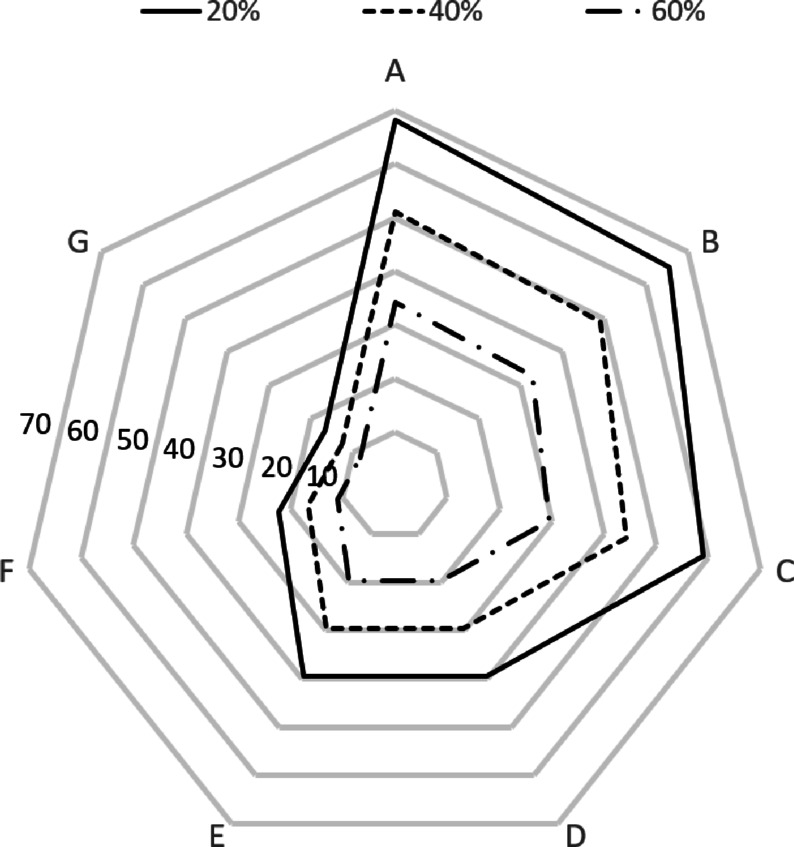
Analysis results (reserve resources).

The change in resources for each specialized center according to the size of the joint resource ratio is shown in Figs. 6 and 7. As the proportion increased, the size of the joint resources increased, and conversely, the size of the remaining joint resources decreased. Specialized centers A, B, and C, which have the largest amounts of resources, showed the greatest changes.

The analysis results are summarized in Tables 7, 8 and 9. First, in the case of a 20% joint utilization resource ratio, the $\:{I}_{td}$ values ​​were 1.28, 1.51, 1.47, and 1.47. The timing of maximum demand and the size of demand were very different for each individual specialty center. This value was the largest in Operational Method 2, indicating the poorest operational stability, and 1 was the smallest, indicating high stability. However, in the case of Method 1, it could not respond to 149.6 PF of usage demand, which can be a very big problem because it corresponds to 71% of the total new demand (210 PF). Method 3 showed the lowest response failure usage demand at 9.5 PF, but had good response ability, and the remaining jointly utilized resources were 60.5 PF, which also had high reserve power. Method 4 had the same $\:{I}_{td}$ value as Method 3, but the demand for use was somewhat higher due to the failure to respond. However, the LF value is 0.51, which means that it uses resources more than twice as efficiently as Method 3. Methods 1 and 2 did not calculate LF values ​​because they assume a situation where there are no jointly utilized resources or when they are used in an integrated manner.

**Table 7:** Analysis results (joint resource ratio: 20%).

	$\:{I}_{td}$	LF	Failure	Reserve resource
1	1.28	-	149.6	0.0
2	1.51	-	22.6	14.9
3	1.47	0.22	9.5	20.2
4	1.47	0.51	14.8	22.0

The analysis results when the joint resource ratio is set to 40% are presented in Table 8. $\:{I}_{td}$ remained unchanged from when the joint utilization ratio was 20%. However, Methods 2 and 3, unlike before, responded to all usage demands, and reserve capacity increased by up to five times. However, in the case of Method 4, the demand for use has slightly increased due to the failure to respond. The scale of reserve resources (84.1–85.8PF) is the size of more than one specialized center, greatly increasing the scope of resource operation. Lastly, LF more than doubled for Method 3 and was at a similar level for Method 4.

**Table 8:** Analysis results (joint resource ratio: 40%).

	$\:{I}_{td}$	LF	Failure	Reserve resource
1	1.28	-	149.6	0.0
2	1.51	-	0.0	84.1
3	1.47	0.43	0.0	84.1
4	1.47	0.50	15.8	85.8

The results of the analysis when the joint resource ratio is set to 60% are presented in Table 9. The results were generally similar to the previous case. However, in Method 4, it was confirmed that the demand for use of the existing 15.8 PF scale due to failure to respond was eliminated, and the scale of reserve resources was improved to 1.8 times.

**Table 9:** Analysis results (joint resource ratio: 60%).

	$\:{I}_{td}$	LF	Failure	Reserve resource
1	1.28	-	149.6	0.0
2	1.51	-	0.0	150.9
3	1.47	0.43	0.0	150.9
4	1.47	0.50	0.0	151.0

**Fig. 8:**
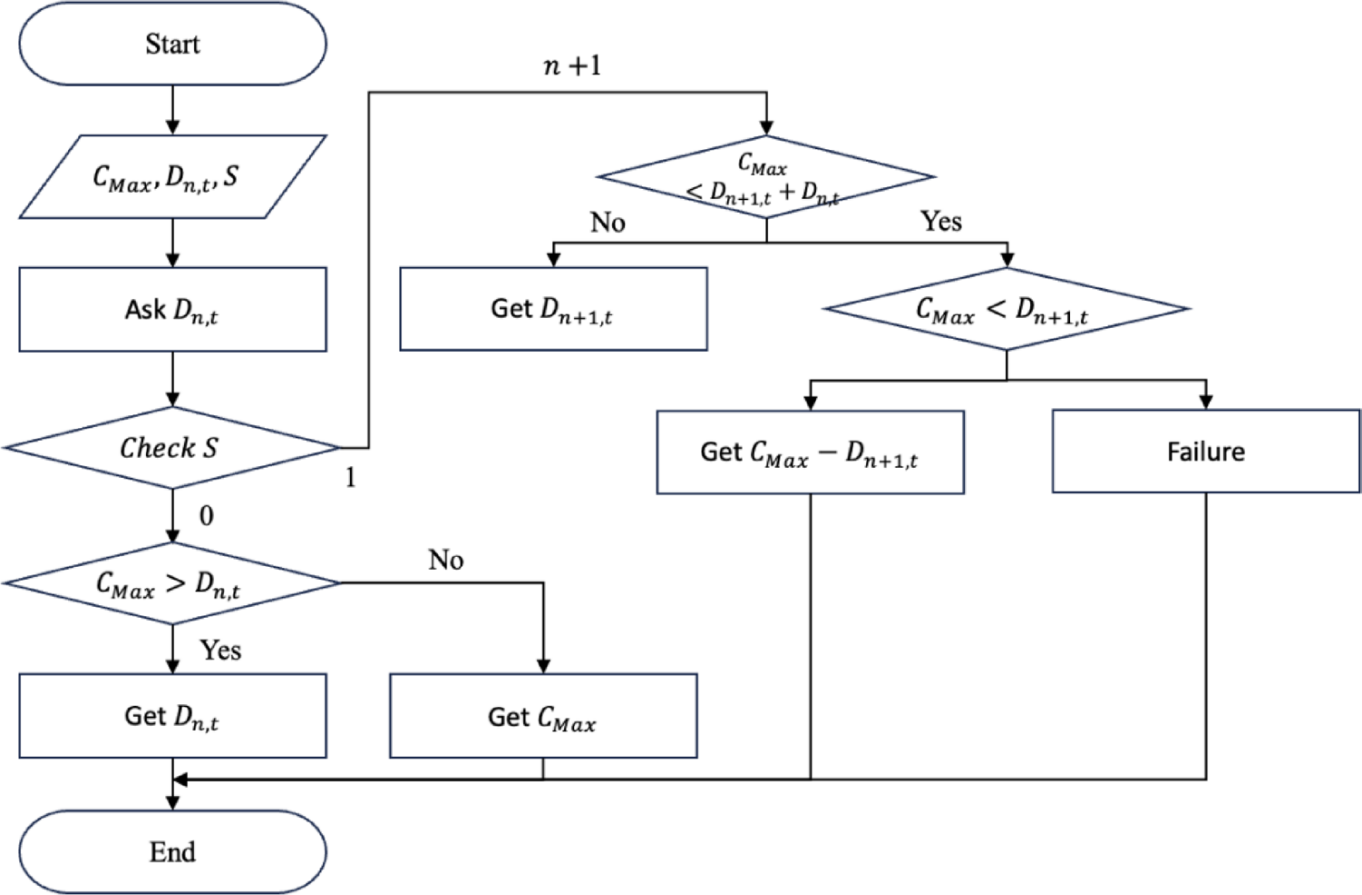
Flowchart of dynamic scheduling algorithm.

So far, an effectiveness evaluation has been conducted on the operational methods applicable to the joint utilization system. Summarizing the analysis results and drawing implications confirmed that methods assessed to be relatively stable or efficient also generate a large amount of reserved resources. This implies that the current method results in a high possibility surplus resource generation, and new technological factors to reduce this must be reflected in the operating method. In the current methods, the next requesting organization can use the resource only after the use time of the organization that requested the resource first has expired. In other words, the organization using the resource has full authority over the requested size of the resource and the usage time, regardless of whether the resource is being used. Thus, it may be considered a “static” scheduling method. However, from the operator’s point of view, even if organization A has the authority to use 100 resources, if unused surplus resources are present, organization A want to let the next organization B use them. If a large number of organizations that want to use resources make some concessions on the timing of use, resources can be provided to all organizations without surplus resources. Owing to that, technical factors related to scheduling are applied to the operational method, and this is called a dynamic scheduling technique in contrast to the existing static scheduling technique. The algorithm for this is shown in Fig. 8. $\:{C}_{Max}$ is the maximum common resource, $\:{D}_{n,t}$ is the n^th^ demand generated at time $\:t$, and $\:n$is an indicator indicating the presence or absence of existing resource-using organizations. If an organization uses common resources at time$\:\:t$, it is marked as 1. If no such organization exists, it is marked as 0.

**Fig. 9:**
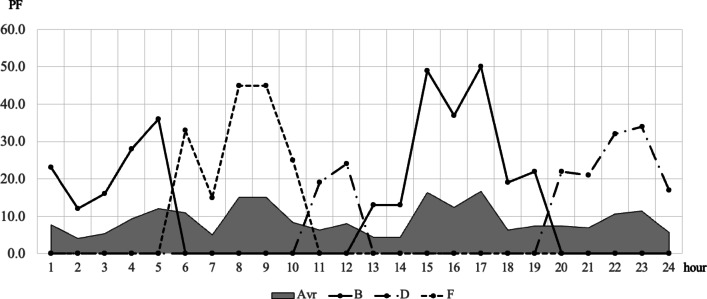
Demand curve by each center (Static).

**Fig. 10:**
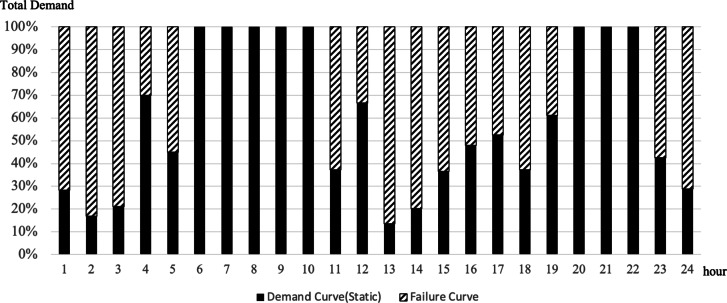
Demand curve for failure (Static).

**Fig. 11:**
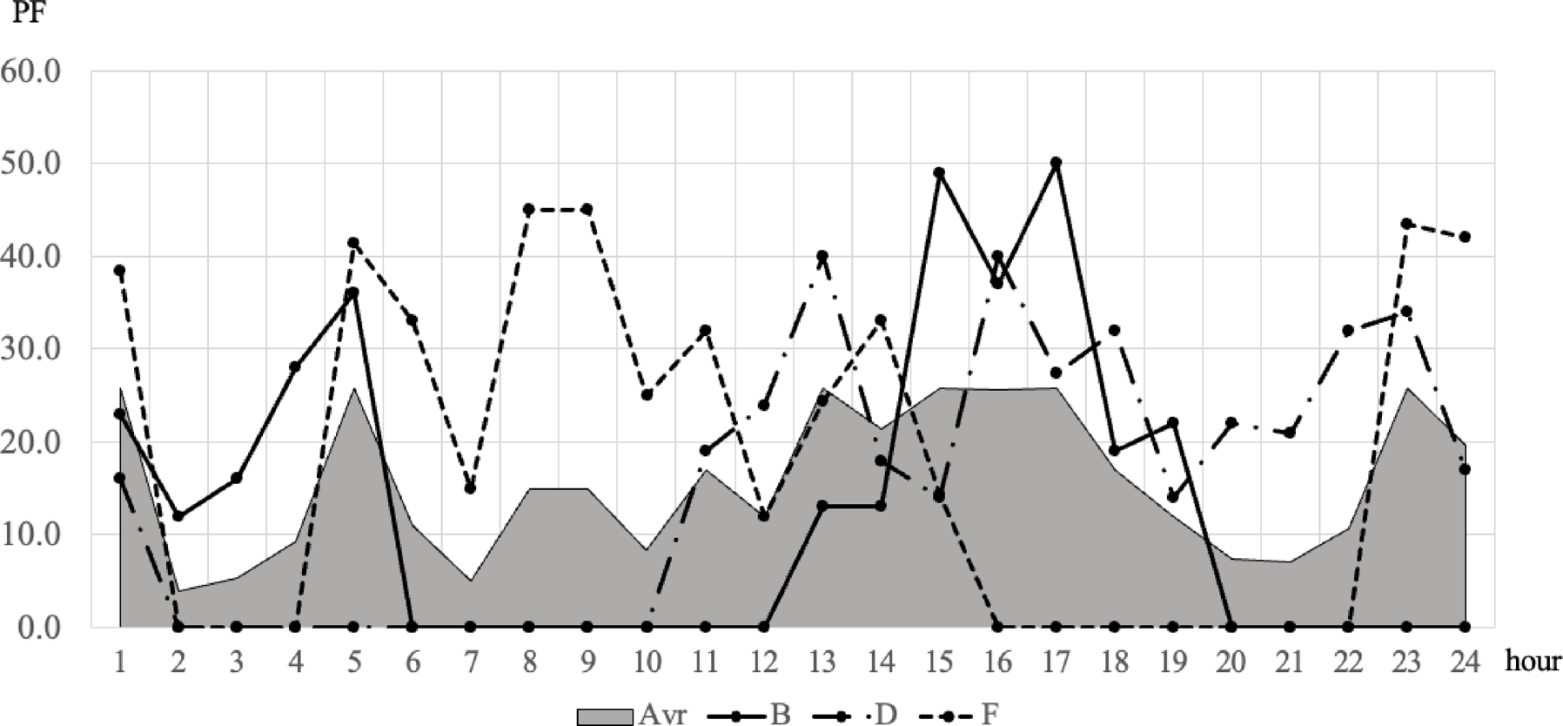
Results of dynamic scheduling curve.

**Fig. 12:**
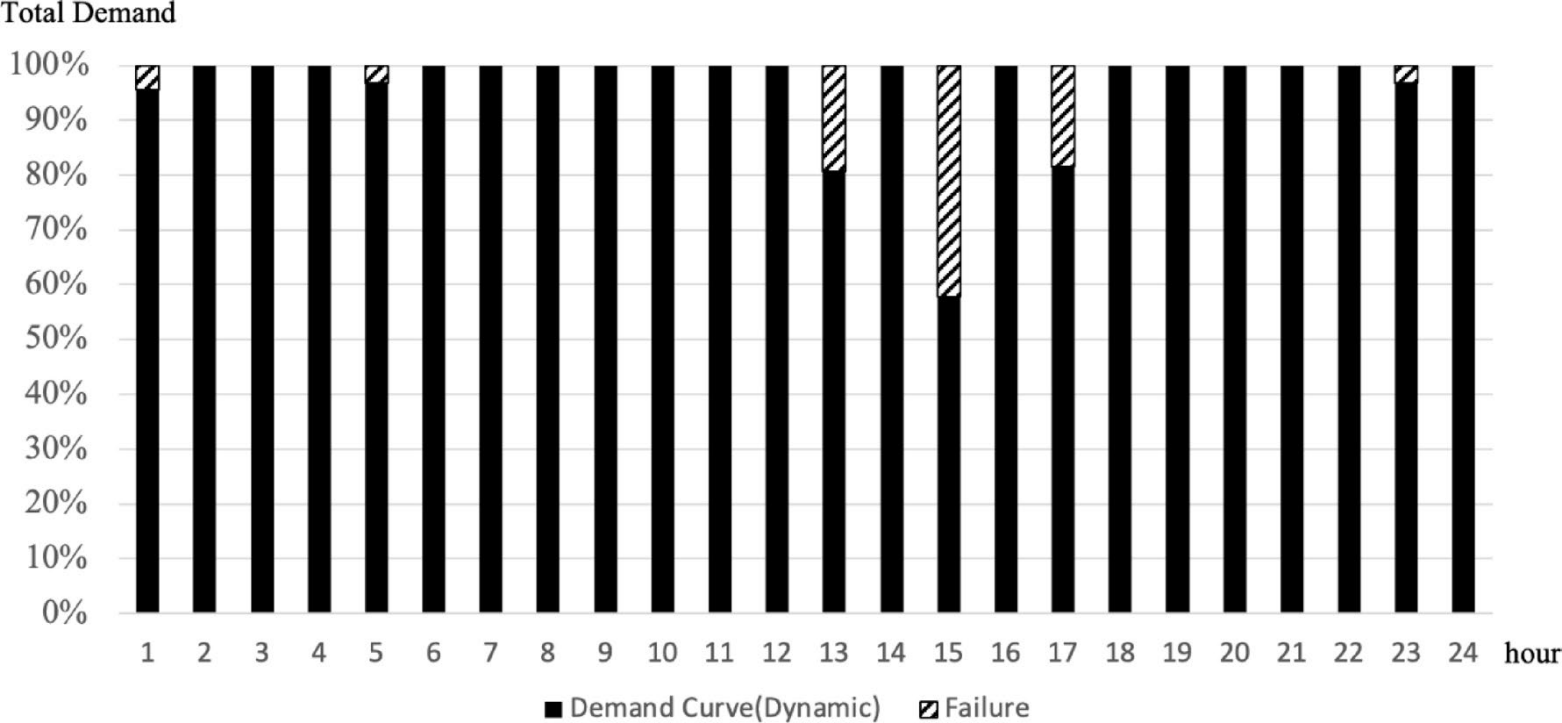
Results of dynamic scheduling failure.

To confirm the effectiveness of applying the scheduling concept, a comparative analysis was conducted between static and dynamic schedule techniques for three organizations, B, D, and F, under the same conditions as in the case study in the previous chapter. These organizations all assumed a situation in which demand for use between 10 and 50 PF occurred randomly, and compared the demand curve for the amount of resources actually used in this situation with the amount of response failure. The comparative analysis results are shown in Figs. 9, 10, 11 and 12. In Figs. 9 and 11, the B, D, and F curves are the demand used by each institution, and $\:Avr$ means the arithmetic average of B, D, and F. In Figs. 10 and 12, the black bar represents the total demand, and the patterned bar represents the demand that failed to respond due to the large amount of demand. Comparing the two methods in Figs. 9 and 11, the static technique had a relatively small $\:Avr$ value. In some sections, a difference of up to 3.5 times existed. Even though the two methods had the same amount of resources, the dynamic method was much larger in terms of the amount of resources provided. Figures 10 and 12 show that in the static method, the amount of resources used was lower than the amount of resources available in each section, and the demand for response failure was also large in the entire section. Response failure: The amount of demand was greater than the amount of resources provided. In contrast, the dynamic method could confirm a low response failure demand, at approximately 8%, showing a significant improvement.

Through comparative analysis of the existing static method and dynamic methods, a method for obtaining the amount of resources required from the supplier side must be determined. However, the importance of the schedule method in satisfying all organizations simultaneously from the user side was identified. Therefore, various factors must be considered in terms of scheduling to manage demand for supercomputer resources, and practical research on the dynamic method presented at the conceptual level in this paper should be conducted.

## Conclusion

This study investigated an integrated operational framework for joint utilization of supercomputing resources in the face of escalating computational demand. Recognizing the limitations of conventional supply-driven allocation models, particularly in multi-institutional environments, we proposed a dynamic, demand-responsive scheduling strategy that leverages empirical data and pricing-based demand elasticity analysis. We began by developing demand models for national and specialized centers using survey-based price elasticity estimates. These models revealed contrasting patterns of user responsiveness, underscoring the need for differentiated management strategies. To evaluate the efficiency of alternative scheduling approaches, we introduced a new system-level index, the Time-Diverse Utilization Index, alongside the conventional Load Factor. Together, these indices enabled a comprehensive assessment of both individual and collective resource efficiency.

Through simulation-based case studies involving seven specialized centers, we compared multiple demand management policies under varying joint resource ratios (20%, 40%, 60%). The results demonstrated that:

- Policy Methods 3 and 4, which enable shared access to pooled resources with prioritization or equal distribution, consistently outperformed other approaches in terms of stability and responsiveness.
- Dynamic scheduling, in contrast to static methods, significantly reduced demand-response failures and surplus idle capacity, thereby improving overall system efficiency.
- The system’s responsiveness improved proportionally with the increase in the joint resource ratio, although this required trade-offs in dedicated resource availability at each center.

These findings offer practical guidance for designing governance mechanisms and scheduling protocols in federated HPC infrastructures. In particular, our proposed evaluation framework provides policymakers and system operators with quantitative tools to balance fairness, efficiency, and flexibility in resource allocation.

This study represents one of the first systematic efforts to apply demand-side management principles to shared supercomputing environments. By adapting concepts from the energy and cloud computing sectors, we offer a novel, empirically grounded approach for supercomputing resource governance.

This research was conducted based on simulated scenarios and assumptions prior to the actual implementation of a national joint utilization system. As such, further empirical validation using real-time operational data is necessary. Future work should focus on: Applying the proposed models to real-world usage data from live supercomputing environments; Exploring adaptive pricing schemes and incentive structures; Integrating machine learning techniques for demand prediction and real-time resource allocation optimization. In addition, we plan to construct a reusable simulation model based on a standardized modeling platform to support reproducible experiments and accelerate comparative analysis of scheduling and reliability strategies across heterogeneous GDN infrastructures. In conclusion, the combination of dynamic scheduling and demand-aware modeling offers a scalable and sustainable path toward managing next-generation supercomputing infrastructures under increasing computational demand.

## Data Availability

The datasets used and/or analysed during the current study are available from the corresponding author on reasonable request.

## Abbreviations

Dynamic Scheduling: A scheduling approach that adjusts resource allocation in real time based on demand fluctuations, system state, and policy rules. It improves responsiveness and resource efficiency compared to static methods
Response Failure: A state in which the available computing resources are insufficient to meet user demand
Elastic Scheduling: A form of dynamic scheduling that allocates resources flexibly based on variations in demand
Static Scheduling: A fixed resource allocation approach where scheduling decisions are predetermined and not adapted at runtime
Joint Utilization Ratio: The proportion of an institution’s computing resources allocated to a shared national or federated pool
